# Survival after Resection of Multiple Tumor Foci of Intrahepatic Cholangiocarcinoma

**DOI:** 10.1007/s11605-019-04184-2

**Published:** 2019-03-18

**Authors:** Stefan Buettner, David W. G. ten Cate, Fabio Bagante, Sorin Alexandrescu, Hugo P. Marques, Jorge Lamelas, Luca Aldrighetti, T. Clark Gamblin, Shishir K. Maithel, Carlo Pulitano, Georgios Antonios Margonis, Matthew Weiss, Todd W. Bauer, Feng Shen, George A. Poultsides, J. Wallis Marsh, Jan N. M. IJzermans, Timothy M. Pawlik, Bas Groot Koerkamp

**Affiliations:** 1 Department of Surgery, Erasmus MC University Medical Center, ‘s Gravendijkwal 230, PO Box 2040, 3000 CA Rotterdam, Netherlands; 2 The Ohio State University Wexner Medical Center, Columbus, OH USA; 3 Fundeni Clinical Institute, Bucharest, Romania; 4 Curry Cabral Hospital, Lisbon, Portugal; 5 Ospedale San Raffaele, Milan, Italy; 6 Medical College of Wisconsin, Milwaukee, WI USA; 7 Emory University, Atlanta, GA USA; 8 University of Sydney, Sydney, Australia; 9 Johns Hopkins Hospital, Baltimore, MD USA; 10 University of Virginia, Charlottesville, VA USA; 11 Eastern Hepatobiliary Surgery Hospital, Shanghai, China; 12 Stanford University, Stanford, CA USA; 13 University of Pittsburgh Medical Center, Pittsburgh, PA USA

**Keywords:** Intrahepatic cholangiocarcinoma, Multiple tumor location, Prognostic staging, Survival

## Abstract

**Background:**

Multiple tumor foci of intrahepatic cholangiocarcinoma (ICC) are often considered a contra-indication for resection. We sought to define long-term outcomes after resection of ICC in patients with multiple foci.

**Methods:**

Patients who underwent resection for ICC between 1990 and 2017 were identified from 12 major HPB centers. Outcomes of patients with solitary lesions, multiple lesions (ML), and oligometastases (OM) were compared. OM were defined as extrahepatic metastases spread to a single organ.

**Results:**

One thousand thirteen patients underwent resection of ICC. On final pathology, 185 patients (18.4%) had ML and 27 (2.7%) had OM. Median survival of patients with a solitary tumor was 43.2 months, while the median survival of patients with 2 tumors was 21.2 months; the median survival of patients with 3 or more tumors was 15.3 months (*p* < 0.001). Five-year survival was 43.3%, 28.0%, and 8.6%, respectively. The median survival of patients without OM was 37.8 months versus 14.9 months among patients with OM (*p* < 0.001); estimated 5-year survival was 39.3% and 10.6%, respectively. In multivariable analysis, the presence of two lesions was not an independent poor prognostic factor for OS (HR 1.19; 95%CI 0.90–1.57; *p* = 0.229). However, the presence of three or more tumors was an independent poor prognostic factor for OS (HR 1.97; 95%CI 1.48–2.64; *p* < 0.001).

**Conclusion:**

Resection of multiple liver tumors for patients with ICC did not preclude 5-year survival: in particular, estimated 5-year OS for resection of two tumors was 28.0%.

**Electronic supplementary material:**

The online version of this article (10.1007/s11605-019-04184-2) contains supplementary material, which is available to authorized users.

## Introduction

Intrahepatic cholangiocarcinoma (ICC) is the second most common primary malignancy of the liver with an incidence of 1–2 per 100,000 persons.^1, 2^ ICC occurs in the bile duct of the peripheral liver parenchyma and often presents late due to the absence of early symptoms.^3^ About 10% of cholangiocarcinomas are ICC.^4, 5^ICC is associated with chronic liver disease secondary to cirrhosis and chronic hepatitis C infection in Western countries.^6^ ICC is also associated with hepatolithiasis, liver fluke infestation, and bile duct malformations such as choledochal cysts.^7–11^ Nevertheless, the underlying liver disease is often not identified and the majority of ICC cases are incidental.

The American Joint Committee on Cancer (AJCC) staging manual is the most commonly used staging model for ICC.^12^ According to the AJCC manual, patients with multiple lesions (ML) or extrahepatic oligometastases (OM) are considered to have less favorable stages and these features often are considered a contra-indication for resection. In fact, the European Association for the Study of the Liver (EASL) in their 2014 guidelines state that ML and OM should be considered relatively strong contraindications to surgery.^13^

Complete resection is the only available curative treatment for ICC, even though it is attainable only in 15–25% of patients.^14–17^ Resection is not without risk, with high perioperative morbidity and mortality associated with (extended) hemihepatectomies.^18–20^ Unresectable ICC is associated with a median survival of only 5 months, which can be prolonged with chemotherapy to 12 months in which 70% of patients experience grade 3 or 4 toxicity.^21–23^ Given the debate regarding how to manage patients with multiple ICC lesions, the current study sought to define long-term outcome after resection of ICC among patients with ML or OM.

## Methods

Patients undergoing resection for ICC between January 1, 1990, and December 31, 2017, were identified from one of 12 participating major hepatobiliary institutions in the USA, Asia, Australia, and Europe (Johns Hopkins University, Baltimore, MD; Emory University, Atlanta, GA; Stanford University Medical Center, Stanford, CA; University of Virginia Health System, Charlottesville, VA; Fundeni Clinical Institute, Bucharest, Romania; Beaujon Hospital, Clichy, France; Curry Cabral Hospital, Lisbon, Portugal; Eastern Hepatobiliary Surgery Hospital, Shanghai, China; Ottowa General Hospital, Ottowa, Canada; Royal Prince Alfred Hospital, Sydney, Australia; San Raffaele Hospital, Milan, Italy; Erasmus MC, University Medical Centre Rotterdam, Rotterdam, the Netherlands). Patients who did not undergo resection, patients who had a macroscopically positive resection margin and patients who received a liver transplantation, were excluded. Only patients with histologically confirmed cholangiocarcinoma were included. Institutional review boards of every participating institution approved this study.

Demographic and clinical data were retrieved from hospital records and included age, sex, BMI, and presence of jaundice. Patient operative risk was estimated using the American Society of Anesthesiologists physical status classification system.^24^ Pathological data such as tumor number, tumor size, major vascular invasion, presence of extrahepatic disease, presence of nodal metastases, final resection margin, and the presence of vascular and/or perineural invasion were also retrieved. Data on treatment-related variables, such as the type of surgery and lymphadenectomy were recorded. A minor hepatectomy was defined as a hepatic resection of less than three Couinaud segments. Margin status was categorized as R0 for tumor negative resection margins and R1 for microscopically positive margins. ML were categorized in two lesions and three or more lesions. In some previous studies, ML have been divided into intrahepatic metastases, lesions at a larger distance from the index tumor or in another segment, and satellite lesions, lesions approximating the index tumor/in the same segment. Because no definitive definition of intrahepatic metastases and satellite lesions exists, we opted to consider both as “multiple lesions”.^25^ OM were defined as metastases limited to a single extrahepatic organ.

Data on short- and long-term outcomes were collected. Short-term outcomes included length of hospital stay (LOS), postoperative morbidity, and mortality. The date of last follow-up and vital status was also collected for all patients. Survival was calculated from the date of index operation. Long-term outcomes were stratified based on multiple lesions and oligometastases.

### Statistical Analysis

Summary statistics were provided as whole numbers and percentages for categorical variables and medians with interquartile range (IQR) for continuous variables. The distribution of categorical variables was tested using the *χ*^2^ test or Fisher’s exact test, as appropriate. The distribution of continuous variables was tested using the Mann–Whitney *U* test. The primary outcome of interest was overall survival (OS), defined as the time interval between the date of surgery and the date of death or last follow-up, as appropriate. Estimates for OS were calculated using the Kaplan-Meier method. Differences in OS were assessed using the Log-Rank test. A multivariable Cox proportional hazards model was used to identify potential risk factors. In the multivariable regression, previously described risk factors, including R1 resection, lymph node metastases, invasion of adjacent organs, and tumor size, were included. Patients with OM were excluded from the multivariable analysis, as metastases that are not resected have such a serious effect on long-term outcomes that these patients were not readily comparable with the other included patients. Results from the Cox proportional hazards model were reported as hazard ratios (HR) and their corresponding 95% confidence intervals (CI). Multiple imputation was used to correct for missing data in the multivariable analysis. All analyses were performed using SPSS 24.0 (IBM, New York) and the *rms* and *mice* packages for R 3.5.1 (https://cran.r-project.org/). All tests were two-sided and *p* < 0.05 defined statistical significance.

## Results

### Cohort Description

In total, 1013 patients were included in this study (Table 1). The median age at resection was 59 years (IQR 50–67), 540 (54.5%) patients were male, and median BMI was 25.4 (22.6–28.2). Preoperative jaundice was present in a minority of patients (*n* = 90, 8.9%). Most patients were classified as ASA II (*n* = 489; 52.0%) or III (*n* = 280; 29.8%) and were treated in the last decade (*n* = 862; 85.1%) with a major surgical procedure (*n* = 593; 58.9%). Distribution across centers for all patients was reported in Supplemental Table 1. ML were more frequently treated in the west and in Australia. OM were resected only in Europe and the USA. Median follow-up after resection was 29.3 months and 507 patients (50.4%) died during follow-up.

**Table 1:** Baseline characteristics

Total	*n* (%)/median (IQR)
	*n* = 1013
Gender	
Male	540 (54.5)
Female	450 (45.5)
Age, years	59 (50–67)
BMI	25.4 (22.6–28.2)
Preoperative jaundice	90 (8.9)
ASA class	
I	103 (11.0)
II	489 (52.0)
III	280 (29.8)
IV	68 (7.2)
Period of treatment	
1990–2000	35 (3.5)
2001–2005	116 (11.5)
2006–2010	411 (40.6)
2011–2017	451 (44.5)
Preoperative chemotherapy	55 (5.4)
Major resection	593 (58.9)
Size, cm	6.2 (4.3–9.0)
Major vascular invasion	100 (10.0)
Microvascular invasion	254 (25.7)
Perineural invasion	149 (16.3)
Extension into adjacent organs	77 (7.7)
R1 resection	128 (12.8)
Lymph node metastases	175 (17.3)
Multiple lesions	
Median number of tumors	2 (2–3)
2 lesions	107 (10.7)
> 2 lesions	78 (7.8)
Oligometastases	
Lung	2 (0.2)
Peritoneum	11 (1.1)
Distant lymph nodes	9 (0.9)
Other	5 (0.5)

The average tumor size was 6.2 cm (IQR: 4.3–9.0). Major vascular invasion was noted in 100 (10.0%) patients; microvascular and perineural invasion was present in 254 (25.7%) and 149 (16.3%) patients, respectively. Direct invasion into adjacent organs was present in 77 (7.7) patients. Multiple tumors were present in 185 patients (18.4%). Patients with ML had a median of two tumors (interquartile range [IQR] 2–3, range 2–11). Oligometastases outside of the liver were present in 27 patients at the time of resection, most of which were located in the peritoneum (*n* = 11; 1.1%) and distant lymph nodes (*n* = 9; 0.9%). These oligometastases were resected in 20/27 patients.

### Number of Tumors

Perioperative outcomes and pathological characteristics were stratified by presence of ML in Table 2. In general, patients with ML had more perioperative complications and more advanced disease at pathological examination. Patients with multiple tumors were more likely to have lymph node metastases (25.4% vs. 15.5%; *p* = 0.001) and were more likely to have disease extension beyond the liver (15.8% vs. 5.9%; *p* < 0.001). ML more often necessitated a major resection (72.4% vs. 55.9%; *p* < 0.001). Postoperative complications were higher in patients with multiple tumors (49.7% vs. 41.8%; *p* = 0.049). Length of stay did not differ across groups. Recurrence occurred in 430 (52.4%) patients with a solitary tumor versus 137 (74.1%) patients with ML (*p* < 0.001).

**Table 2:** Postoperative outcomes stratified by intrahepatic metastases

Variable	Single tumor (*n* = 821)	Multiple tumors (*n* = 185)	*p* value*
Preoperative chemotherapy	40 (4.9)	15 (8.1)	0.080
R1 margin	97 (11.9)	29 (15.8)	0.154
Lymph node metastases	127 (15.5)	47 (25.4)	0.001
Oligometastases	20 (2.4)	7 (3.8)	0.314
Direct invasion other organ	48 (5.9)	29 (15.8)	< 0.001
Perineural invasion	118 (15.9)	30 (18.0)	0.505
Major vascular invasion	76 (9.3)	23 (12.5)	0.189
Major resection	458 (55.9)	131 (72.4)	< 0.001
Postoperative complication	343 (41.8)	92 (49.7)	0.049
Clavien–Dindo grade			0.036
I-II	207 (60.0)	44 (47.8)	
IIIa-V	138 (40.0)	48 (52.2)	
90-day postoperative mortality	50 (6.1)	11 (5.9)	0.941
Length of stay, days	12 (7–17)	12 (7–18)	0.668
Recurrence	430 (52.4)	137 (74.1)	< 0.001

*Fisher’s exact test was used for categorical variables with expected counts < 5

Patients with OM, like patients with ML, were diagnosed with worse prognostic factors and had worse perioperative outcomes (Table 3). In particular, patients with OM were more likely to have R1 margins (34.6% vs. 12.2%; *p* = 0.003), lymph node metastases (55.6% vs. 16.3%; *p* < 0.001), and invasion outside of the liver (48.1% vs. 6.6%; *p* < 0.001). Complications occurred more frequently in patients with OM (70.4% vs. 42.7%; *p* = 0.004). Postoperative mortality was also much higher than in patients without oligometastases (22.2% vs. 5.6%, respectively; *p* = 0.004). There was no significant difference in recurrence (63.0% vs. 56.0%; *p* = 0.472).

**Table 3:** Postoperative outcomes stratified by the presence of oligometastases

Variable	No oligometastases (*n* = 982)	Oligometastases (*n* = 27)	*p* value*
Preoperative chemotherapy	52 (5.3)	3 (11.1)	0.178
R1 margin	119 (12.2)	9 (34.6)	0.003
Lymph node metastases	160 (16.3)	15 (55.6)	< 0.001
Multiple lesions	178 (18.2)	7 (25.9)	0.314
Direct invasion other organ	64 (6.6)	13 (48.1)	< 0.001
Perineural invasion	143 (16.2)	5 (19.2)	0.596
Major vascular invasion	95 (9.7)	5 (18.5)	0.179
Major resection	571 (58.4)	20 (74.1)	0.104
Postoperative complication	419 (42.7)	19 (70.4)	0.004
Clavien–Dindo Grade			0.691
I-II	241 (57.2)	10 (52.6)	
IIIa-V	180 (42.8)	9 (47.4)	
90-day postoperative mortality	55 (5.6)	6 (22.2)	0.004
Length of stay, days	12 (7–17)	13 (9–20)	0.197
Recurrence	550 (56.0)	17 (63.0)	0.472

*Fisher’s exact test was used for categorical variables with expected counts < 5

### Survival Estimates

**Fig. 1:**
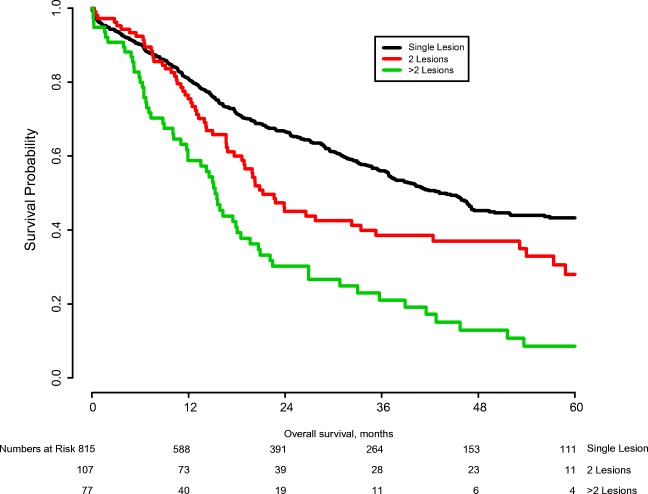
Overall survival stratified by presence of intrahepatic metastases (*p* < 0.001)

**Fig. 2:**
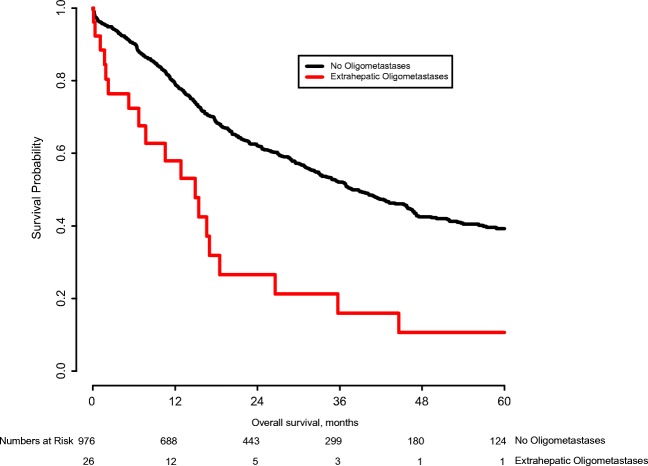
Overall survival stratified by presence of oligometastases (*p* < 0.001)

Overall survival was compared among patients with and without multiple lesions (Fig. 1). Median OS of patients with two tumors was 21.2 months and median OS of patients with three or more tumors was 15.3 months, while patients with only a single tumor had a median OS of 43.2 months (*p* < 0.001). At 5 years follow-up, 28.0% of patients with two tumors were still alive vs. 43.3% of patients with a single tumor. A similarly large difference was observed in median OS between patients with and without OM (14.9 months vs. 37.8 months; *p* < 0.001; Fig. 2). Five-year survival for patients with OM was 10.6% versus 39.3 for patients without OM.

In multivariable analysis (Table 4), known risk factors for early mortality in patients with ICC were included. Factors significantly associated with survival included R1 resection margin (HR 1.48; 95%CI 1.12–1.95; *p* = 0.005), lymph node metastases (HR 1.88; 95%CI 1.48–2.39; *p* < 0.001), invasion into adjacent organs (HR 1.63; 95%CI 1.17–2.29; *p* = 0.004), and size in centimeter (HR 1.05; 95%CI 1.02–1.07; *p* < 0.001). The presence of two lesions was not an independent poor prognostic factor for OS (HR 1.19; 95%CI 0.90–1.57; *p* = 0.229). However, the presence of three or more tumors was an independent poor prognostic factor for OS (HR 1.97; 95%CI 1.48–2.64; *p* < 0.001).

**Table 4:** Multivariable survival analysis

Variable	Hazard ratio	95% CI	*p* value
Age, years	1.00	1.00–1.01	0.417
ASA III/IV	1.05	0.86–1.28	0.636
R1 margin	1.48	1.12–1.95	0.005
Lymph node metastases	1.88	1.48–2.39	< 0.001
Direct expansion other organ	1.63	1.17–2.29	0.004
Perineural invasion	1.18	0.89–1.56	0.256
Major vascular invasion	1.04	0.76–1.43	0.793
Size, cm	1.05	1.02–1.07	< 0.001
Lesion number			
Single lesion	Ref	–	–
2 lesions	1.19	0.90–1.57	0.229
> 2 lesions	1.97	1.48–2.64	< 0.001

## Discussion

In this study of more than 1000 patients who underwent curative resection for ICC, the number of tumors had a large impact on the median OS. Specifically, OS was 43.2 months for solitary tumors, 21.2 months for two tumors, and 15.3 months for three or more tumors (*p* < 0.001). However, resection of multiple tumors did not preclude 5-year survival as the estimated 5-year OS for resection of two tumors was 28.0%. Previously, multiple tumor foci have been considered a relative contra-indication for resection in guidelines.^12, 13, 26^ In comparison, median OS for systemic chemotherapy and locoregional ablative treatments (e.g., radio-embolization) rarely exceeds 12 months.^23, 27–32^In the current study, on multivariable analysis, the presence of more than two lesions was an independent poor prognostic factor, while the presence of two lesions was not.

Although a comprehensive definition of intrahepatic metastases does not exist, intrahepatic metastases of ICC are most commonly defined as tumor processes at larger distances, e.g., 2 cm from the index tumor, or in another Couinaud segment of the liver.^33, 34^ It is currently insufficiently understood, whether satellite lesions, tumors within the same segment and close to the index tumor, have a separate natural history from intrahepatic metastases.^25^ Theoretically, tumors at larger distance would have a larger impact on prognosis because these lesions may represent hematogenous intrahepatic dissemination.^25, 35^ The use of Couinaud segments, a model for macroscopic liver anatomy, seems arbitrary, as it has no basis in physiology or carcinogenesis.^36^ Two recent smaller-scale studies demonstrate the possibility for long-term survival for both intrahepatic metastases and satellite lesions.^37, 38^ However, only in one of these could a rather small difference in survival between satellites and distant liver metastases be demonstrated.^37^ Because of these reasons, we opted to consider lesions of both categories as “multiple lesions.”

Many small studies have attempted to evaluate clinical outcomes after ML and a systematic review has confirmed the gravity of this prognostic factor.^39–41^ Mostly, studies confirm that there is a correlation between ML and prognostic factors of advanced disease, such as lymph node metastases, vascular involvement, and distant metastases. Because of the relative rarity of ICC, however, whether ML are an absolute contra-indication for surgery, especially in absence of other aggravating factors, remains unclear. In this large multi-institutional study, enough statistical power was available to account for confounding factors in multivariable analysis. As such, we were able to confirm the prognostic importance of ML, with a median difference in survival of 21 months for two tumors and 15 months for three or more tumors in univariable analysis. In addition, patients with ML were shown to have a higher likelihood of lymph node metastases, direct invasion into adjacent organs, and necessity of a major resection. Of note, after correcting for possible confounding factors, ML were not a significant prognostic factor in multivariable analysis, and the estimated difference in survival in a cohort without other risk factors was minimal.

Local techniques for management of ICC include hepatic arterial infusion, TACE, and chemo-embolization. These techniques have in common that they rely on the dual blood supply of the liver.^42–44^ Hepatic arterial infusion therapy works by continuous infusion of floxuridine directly into the hepatic artery. A study based on two prospective trials suggests 5-year survival can be as high as 20%.^45^ In similar studies, TACE has been observed to have a 3-year survival of 15%.^42, 46^ Radio-embolization has an observed 3-year survival of 15%.^42^ The results of this study indicate that in well-selected patients with ML, superior results can be achieved with complete resection. Because of the minimally invasive nature of local techniques, the comorbidity after application is lower than for resection. Even using the latest techniques, oncologic liver resection has a reported comorbidity of 30–50% and a mortality of up to 3–5%.^18, 47^ In this study, the postoperative complications and mortality were more common in patients with ML. Better long-term survival combined with higher postoperative complications necessitated a strict selection of patients with ML for surgical resection.

Like ML, a definitive definition for oligometastases does not exist and the literature on this subject is scarce. In this study, we defined OM as spread to one extrahepatic organ. Even with this definition, only 27 patients were identified, 20 of whom also underwent resection of these metastases. Long-term outcome after survival was poor, with only an estimated 10% of patients surviving to 5 years after resection. Like intrahepatic metastases, OM correlated with other predictors of poor survival, such as lymph node metastases, a positive resection margin and direct growth into neighboring organs. Postoperative outcomes after resection were significantly worse in patients with OM. These poor perioperative outcomes, combined with a grave prognosis indicate that restraint should be exercised when deciding to resect OM. More, ideally, prospective data is necessary for evaluating the advantages and drawbacks of surgery for this patient population.

This study has several strengths and weaknesses. To our knowledge, this is the largest available study assessing the long-term outcomes of surgery for ML and OM. This made it possible to perform multivariable analyses, leading to more precise estimates of survival correlation. Because no follow-up protocol was in place, we have opted to limit ourselves to the more objective OS as an outcome. The multicentricity of this study made the data presented herein more broad applicability worldwide. Even so, due to the small number of patients with oligometastases, we were not able to fully determine the subgroup of patients that would benefit from a surgical resection. Apart from oncologic characteristics, treatment choices such as preoperative chemotherapy and other liver-directed therapies could have large impact on survival. In this retrospective review, we cannot possibly reconstruct all treatment choices, making a large-scale prospective study especially suited for a more detailed analysis of the preoperative course. The result of this is the main weakness of this study: selection bias. Included patients who underwent resection for multiple intrahepatic tumors were part of a highly selected cohort. Although difficulty in patient selection remains, this study offers a credible case for not treating ML as an absolute contra-indication. Finally, in our database, we had insufficient data to accurately differentiate between intrahepatic metastases and satellite lesions.

## Conclusion

Complete resection of multiple tumors should be considered in selected ICC patients, especially in the presence of two tumors.

## Electronic Supplementary Material

ESM 1 (DOCX 18 kb)
