# Supplementary material for: Survival after Resection of Multiple Tumor Foci of Intrahepatic Cholangiocarcinoma
Source: J Gastrointest Surg. 2019 Mar 18;23(11):2239–46. doi: 10.1007/s11605-019-04184-2 (PMC6831534; doi:10.1007/s11605-019-04184-2)
Supplement: Supplementary file 1 — (DOCX 18 kb) [file 11605_2019_4184_MOESM1_ESM.docx]

| Supplemental Table 1. Treatment center stratified by multiple lesions and oligometastases | | | | |  | |  | |  |
| --- | --- | --- | --- | --- | --- | --- | --- | --- | --- |
| Variable | Single Tumor  (n = 821) | Multiple Tumors  (n = 185) | p-value | No oligometastases (n = 982) | | Oligometastases (n = 27) | | p-value | |
| Center |  |  | <0.001 |  | |  | | <0.001 | |
| 1 | 69 (8.4) | 45 (24.3) |  | 105 (10.7) | | 10 (37.0) | |  | |
| 2 | 56 (6.8) | 17 (9.2) |  | 73 (7.4) | | 0 (0.0) | |  | |
| 3 | 39 (4.8) | 10 (5.4) |  | 50 (5.1) | | 1 (3.7) | |  | |
| 4 | 35 (4.3) | 14 (7.6) |  | 47 (4.8) | | 2 (7.4) | |  | |
| 5 | 290 (35.3) | 22 (11.9) |  | 312 (31.8) | | 0 (0.0) | |  | |
| 6 | 19 (2.3) | 2 (1.1) |  | 22 (2.2) | | 0 (0.0) | |  | |
| 7 | 59 (7.2) | 35 (18.9) |  | 89 (9.1) | | 5 (18.5) | |  | |
| 8 | 71 (8.6) | 4 (2.2) |  | 70 (7.1) | | 6 (22.2) | |  | |
| 9 | 69 (8.4) | 15 (8.1) |  | 82 (8.4) | | 1 (3.7) | |  | |
| 10 | 23 (2.8) | 0 (0.0) |  | 23 (2.3) | | 0 (0.0) | |  | |
| 11 | 34 (4.1) | 15 (8.1) |  | 49 (5.0) | | 0 (0.0) | |  | |
| 12 | 57 (6.9) | 6 (3.2) |  | 60 (6.1) | | 2 (7.4) | |  | |
| Continent of origin |  |  | <0.001 |  | |  | | <0.001 | |
| Europe | 295 (35.9) | 100 (54.1) |  | 374 (38.1) | | 24 (88.9) | |  | |
| North America | 202 (24.6) | 48 (25.9) |  | 247 (25.2) | | 3 (11.1) | |  | |
| Asia/Oceania | 324 (39.5) | 37 (20.0) |  | 361 (36.8) | | 0 (0.0) | |  | |
